# Berberine Inhibits Dengue Virus through Dual Mechanisms

**DOI:** 10.3390/molecules26185501

**Published:** 2021-09-10

**Authors:** Thippayawan Ratanakomol, Sittiruk Roytrakul, Nitwara Wikan, Duncan R. Smith

**Affiliations:** 1 Institute of Molecular Biosciences, Mahidol University, Nakhon Pathom 73170, Thailand; thippayawan.raa@student.mahidol.ac.th; 2 National Center for Genetic Engineering and Biotechnology, National Science and Technology Development Agency, Rangsit 12120, Thailand; sittiruk@biotec.or.th

**Keywords:** antiviral activity, berberine, isoquinoline alkaloid, dengue virus, Zika virus, chikungunya virus

## Abstract

Mosquito transmitted viruses, particularly those of the genus *Flavivirus*, are a significant healthcare burden worldwide, especially in tropical and sub-tropical areas. However, effective medicines for these viral infections remains lacking. Berberine (BBR) is an alkaloid found in some plants used in traditional medicines in Southeast Asia and elsewhere, and BBR has been shown to possess anti-viral activities. During a screen for potential application to mosquito transmitted viruses, BBR was shown to have virucidal activity against dengue virus (DENV; IC_50_ 42.87 µM) as well as against Zika virus (IC_50_ 11.42 µM) and chikungunya virus (IC_50_ 14.21 µM). BBR was shown to have cellular effects that lead to an increase in cellular DENV E protein without a concomitant effect on DENV nonstructural proteins, suggesting an effect on viral particle formation or egress. While BBR was shown to have an effect of ERK1/2 activation this did not result in defects in viral egress mechanisms. The primary effect of BBR on viral production was likely to be through BBR acting through AMPK activation and disruption of lipid metabolism. Combined these results suggest that BBR has a dual effect on DENV infection, and BBR may have the potential for development as an anti-DENV antiviral.

## 1. Introduction

The global threat from arthropod-borne viruses (arboviruses) has become more serious due to dispersion of these viruses across continents. This is especially true for dengue virus (DENV; family *Flaviviridae*, genus *Flavivirus*) which has become distributed across more than 100 tropical and subtropical countries [1]. Other viruses in the *Flavivirus* genus that cause high hospitalizations and morbidity along with DENV include West Nile virus (WNV), yellow fever virus (YFV) and Japanese encephalitis virus (JEV) [2]. To combat these viruses there are only two safe and effective vaccines available for JEV and YFV [3], while an approved DENV vaccine has concerns with regards to both safety and efficacy [4,5]. There are currently no specific approved antivirals towards any mosquito transmitted virus.

Transmission of DENV is predominantly by *Aedes* spp. mosquitoes [6]. The DENV virion is a spherical shaped enveloped virus, with surface proteins arranged in icosahedral symmetry containing a positive-sense single stranded RNA genome of approximately 11 kb [7]. The genomic RNA has a single open reading flame (ORF) that serves as a template to encode a single polyprotein that later undergoes enzymatic cleavage into three structural (E, prM/M, C) and seven non-structural (NS) proteins (NS1, NS2A, N2B, NS3, NS4A, NS4B, NS5) [8].

DENV is in global concern as half of the world population are at risk of infection, and an estimated 400 million infections occur annually, resulting in some 100 million cases that show some symptoms [1]. Where symptomatic, DENV infection generally results in relatively mild symptoms including fever, headache, nausea and a rash, although in some cases the disease can progress to more severe conditions including dengue hemorrhagic fever and the life-threatening dengue shock syndrome [6]. In the absence of a specific antiviral agent, treatment is only supportive. In Thailand, DENV is an annual threat to the Thai population, and a significant challenge to national health care as the number of cases can range to up to 150,000 cases per year [9].

Berberine (BBR) is an isoquinoline alkaloid (Supplemental Figure S1) that can be extracted from several plants such as *Tinospora crispa* and *Berberis vulgaris*, and these plants have long been used in Ayurvedic, Chinese and Southeast Asian (including Thailand) traditional remedies [10,11]. BBR has been shown to have pharmacological activity in various diseases including inflammatory disorders, metabolic syndromes, infections and cancers [12,13,14]. In term of infectious diseases, BBR has been shown to be an effective antiviral agent against several viruses including those belonging to the genus *Flavivirus*. Several mechanism of actions for BBR have been proposed including BBR acting through the down-regulation of proteins in cell communication cascades such as MAPK and Akt in chikungunya virus (CHIKV), enterovirus 71 and respiratory syncytial virus infections [15,16,17,18], and through inhibition of hepatitis C virus entry by possibly interacting with the viral protein E2 [19]. Studies have also shown that BBR can reduce Zika virus (ZIKV) infection in male germ cells [20]. Some studies have shown that BBR can be useful in the treatment of dyslipidemia [21,22,23], and given the proposed importance of lipids and lipid metabolism in *Flavivirus* replication and virion assembly [24,25,26,27], this might represent an alternative mechanism of action for BBR.

In this study, we investigated the antiviral activity of BBR towards DENV by examining direct virucidal activity, as well as investigating any effects on viral productivity and infectivity. We show that BBR exerts a direct virucidal effect, as well as reducing new virion production through a post-replication mechanism. Reduced virus production, coupled with accumulation of viral E protein in response to BBR treatment suggests that BBR interferes with viral assembly/egression. With a dual mechanism of action, BBR has the potential for further development as a therapeutic agent to treat DENV infection.

## 2. Results

### 2.1. Virucidal Activity of BBR

**Figure 1:**
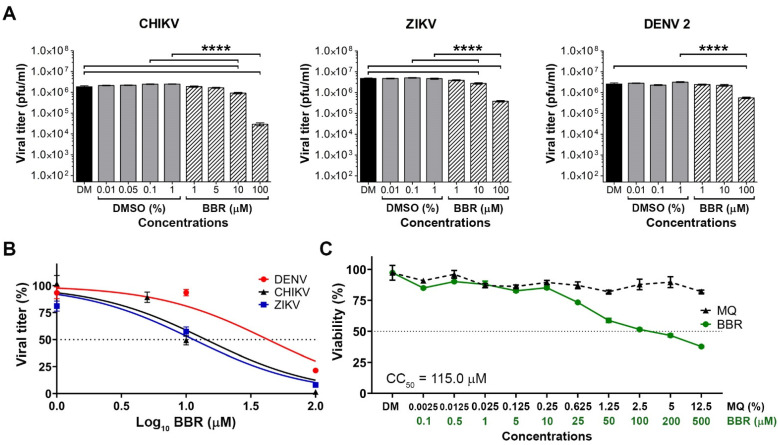
Direct effect of BBR towards viruses and cells. (**A**) Stock CHIKV, ZIKV and DENV 2 were treated with different concentrations of BBR or equivalent concentration of vehicle (DMSO) for 1 h before determining viral titer by plaque assay. Experiment was undertaken independently in triplicate with duplicate plaque assay. (**B**) Viral titers from (**A**) were used to determine IC_50_ values. (**C**) BHK-21 cells were treated with different concentration of BBR or equivalent volume of MQ water in DMEM for 24 h before cell viability was determined by the MTT assay. Experiment was undertaken as 4 independent replicates. Errors bars show ± S.E.M. **** *p* ≤ 0.0001.

To evaluate the potential of BBR as an antiviral agent with potential activity against DENV, we first established whether BBR had direct virucidal activity. We additionally determined whether BBR had virucidal activity against two other arboviruses, ZIKV and CHIKV. Stock DENV 2, CHIKV and ZIKV were therefore incubated for 1 h with various concentrations of BBR, or the equivalent volume of vehicle. After this time the virus titer was determined by plaque assay. The results showed that all viruses were affected by high concentration of BBR, which resulted in at least 1 log reduction in titer when virus was incubated with 100 µM BBR (Figure 1A). The viral titers were used to determine the IC_50_ values (Figure 1B) and the lowest IC_50_ calculated was for ZIKV (11.42 µM), followed by CHIKV (14.21 µM) and DENV 2 (42.87 µM).

### 2.2. Cytotoxicity of BBR

The cytotoxicity of BBR towards BHK-21, A549, HEK293T/17, Huh-7 and HepG2 cells was determined using the MTT assay. BBR concentrations ranging from 0.1–500 µM were used to treat cells for 24 h in parallel with cells treated with an equivalent final volume of vehicle in complete medium as a control. The results showed a degree of cell type variability in cytotoxicity, with CC_50_ values of 115 µM (BHK-21), 79.54 µM (A549), 8.24 µM (HEK293T/17), 70.98 µM (Huh-7) and 244.1 µM (HepG2) (Figure 1C, Supplemental Figure S2). In addition to the MTT assays, the cell morphology of BHK-21 was also examined over a range of BBR concentrations, and this showed that the maximal concentration of BBR in this cell type that showed no morphological changes was 25 µM (Supplemental Figure S3).

### 2.3. Effect of BBR on DENV 2 Infection

As a previous study on the antiviral effects of BBR to CHIKV was undertaken in BHK-21 cells [17], this cell line was selected to determine whether BBR had cellular effects on DENV replication, in addition to the direct virucidal effects. Prior to this, an appropriate MOI was determined, by infecting BHK-21 cells with different MOI of DENV 2, and the level of infection determined by flow cytometry. The results showed that an MOI of 2 was optimal (Supplemental Figure S4), and this was used for all experiments.

**Figure 2:**
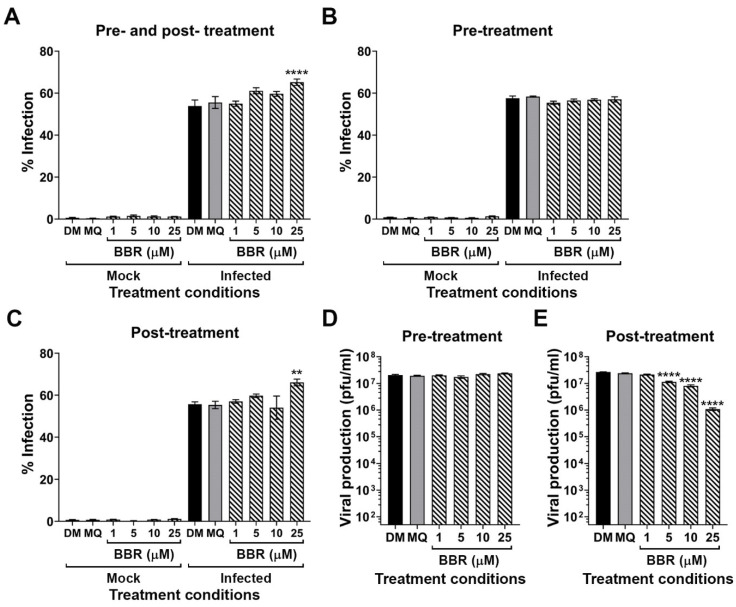
Effect of time of addition of BBR on DENV infection. BHK-21 cells were treated with an equivalent volume of DMEM (DM), milli-Q water (MQ) or 1–25 µM BBR before and/or after being mock infected or DENV 2 infected for 2 h follow by removal of media. Infected cells were further incubated for another 24 h with or without BBR as appropriate before collection of cells and supernatants. (**A**–**C**) Cells were analyzed for level of infection by flow cytometry. (**D**,**E**) DENV 2 titer was determined in the supernatant of experiments in which cells were infected. All experiments were undertaken independently in triplicate, with duplicate plaque assay. Error bars show ± S.E.M. ** *p* ≤ 0.01, **** *p* ≤ 0.0001.

To determine the effect of BBR on DENV 2 infection, three BBR treatment protocols were used, namely treatment of cells both pre-and post-infection, treatment of cells post-infection only and treatment of cells pre-infection only. The result showed that BBR significantly increased levels of DENV 2 infection with treatment at the highest BBR dose of 25 µM, under both pre-and post-, and post-only treatment regimens (Figure 2A,C), while pre-treatment of cells (prior to infection) had no effect on the level of DENV 2 infection (Figure 2B).

Supernatants were collected from the individual pre-treatment and post-treatment experiments, and assayed to determine viral titer by plaque assay. Pre-treatment of cells with BBR had no effect on virus titer (Figure 2D), while post-treatment showed a dose dependent reduction in virus titer (Figure 2E). The half-maximal effective concentration (EC_50_) was 3.66 µM based on assay of post-treatment viral productivity (Supplemental Figure S5). The selectivity index (SI) determined from CC_50_/EC_50_ was 31.42.

**Figure 3:**
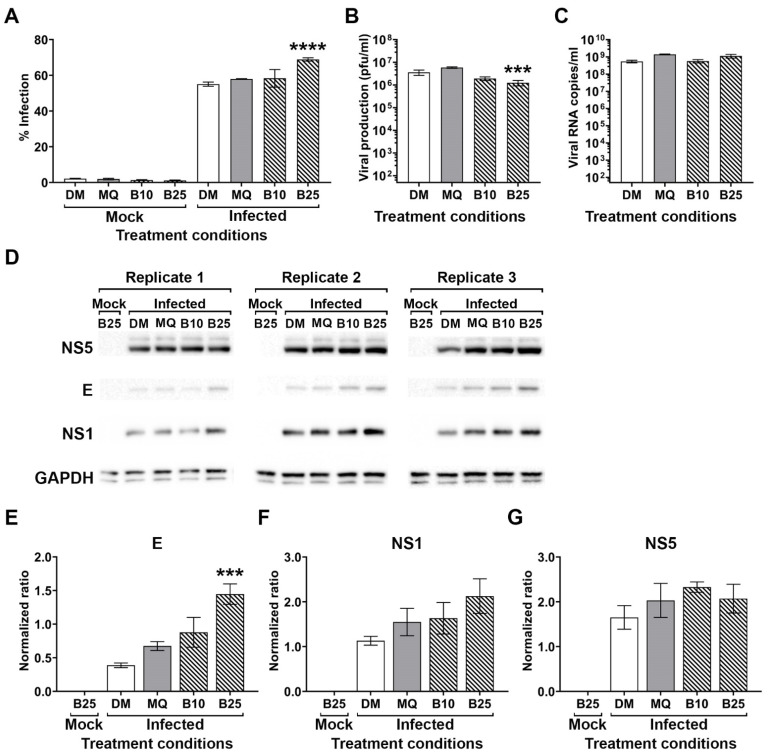
Effect of BBR on DENV infection. BHK-21 cells were mock or DENV 2 infected before being treated with an equivalent volume of DMEM, MQ water or 10 or 25 µM of BBR for 24 h. After 24 h cells and supernatants were collected. (**A**) Level of infection was determined by flow cytometry. (**B**) DENV 2 titer in the supernatant was determined by plaque assay. (**C**) Level of genome in cells was determined by quantitative real-time RT-PCR. (**D**) Levels of DENV NS5, E and NS1 proteins were determined by western blot in parallel with host cell GAPDH as a control. (**E**–**G**) Band intensities from (**D**) were quantitated and normalized to expression of GAPDH. All experiments were undertaken independently in triplicate, with duplicate plaque assay. Error bars show ± S.E.M. *** *p* ≤ 0.001, **** *p* ≤ 0.0001).

To confirm the result, we repeated the post-treatment experiment, and this time determined the levels of the DENV genome and viral protein expression (NS5, E and NS1), as well as viral titer and level of infection. The results (Figure 3) confirmed that BBR treatment increased the level of infection (Figure 3A), while reducing the level of viral production (Figure 3B). The level of DENV genome was slightly, but not significantly, increased by BBR treatment (Figure 3C). Western blot analysis showed a significant increase of DENV E protein, with no significant increase in NS1 or NS5 protein expression (Figure 3D, with quantitation in Figure 3E–G) although the level of NS1 was also slightly but not significantly elevated. Collectively, these results can be interpreted to suggest that BBR induces either a deficit in viral egress, or in formation of the DENV virion prior to egress.

### 2.4. Influence of BBR on the Secretory Pathway

**Figure 4:**
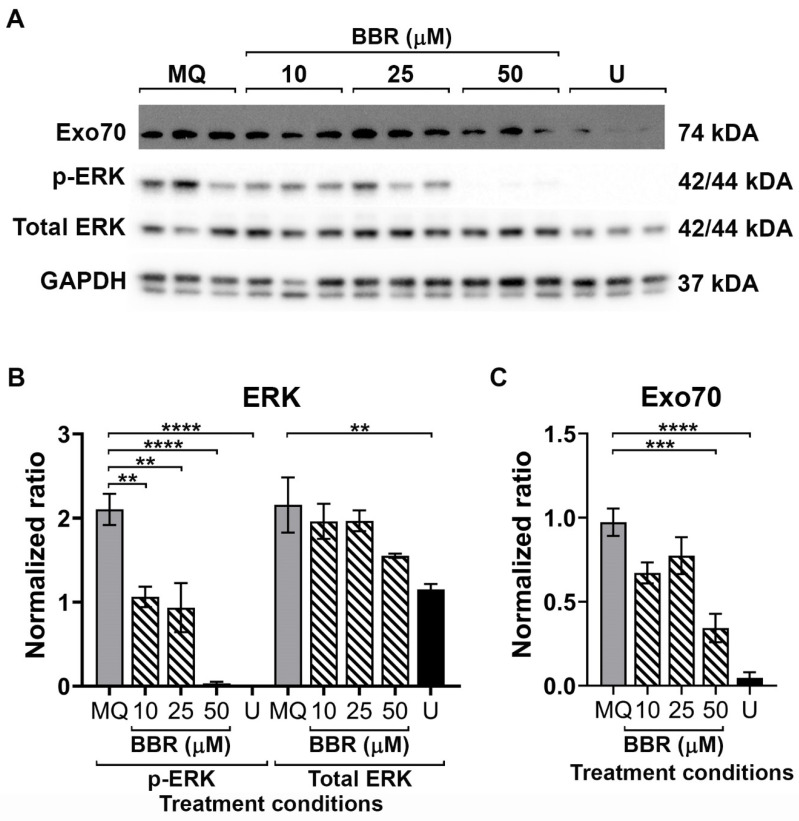
Effect of BBR on Erk1/2 and Exo70. BHK-21 cells were either treated with vehicle, U0126 or BBR at 10, 25 or 50 µM for 24 h, after which cells were collected and proteins prepared. (**A**) Expression levels of Exo70, p-Erk1/2, total Erk and GAPDH were determined by western blotting. (**B**,**C**) Band intensities from (**A**) were quantitated and normalized against GAPDH. Experiment was undertaken independently in triplicate. Error bars show ± S.E.M. ** *p* ≤ 0.01 *** *p* ≤ 0.001, **** *p* ≤ 0.0001.

Exo70 is a protein subunit of the exocyst complex which plays a crucial role in cellular trafficking and secretion, and it has previously been shown that Exo70 is directly involved with DENV secretion from cells [28]. Exocyst complex assembly can be directly activated by phosphorylation of the extracellular signal-regulated kinase (ERK1/2, p42/p44 MAPK) [28,29]. To determine whether BBR was exerting an effect on the exocyst complex, cells were treated with BBR and presence of Exo70 and the phosphorylation status of ERK determined by western blotting. In addition, cells were treated with U0126, an inhibitor of MEK, the upstream kinase of ERK [30]. Results (Figure 4) showed that BBR did indeed reduce the phosphorylation of ERK without significantly affecting the level of total ERK protein, but this was not reflected in a significant reduction in levels of Exo70. In contrast, treatment of cells with U0126 resulted in a significant reduction in both ERK1/2 phosphorylation (without any significant effect on total protein levels), as well as a significant reduction in Exo70 levels (Figure 4). This suggests that BBR is not exerting its major effect through inhibition of DENV egress from cells. In this experiment, cells were also treated with 50 µM BBR, which showed a highly significant reduction in phospho-ERK1/2 and levels of Exo70, however because of concerns that this resulted from cytotoxicity at this concentration of BBR, the result was not further interpreted.

### 2.5. BBR Affects Lipid Metabolism via AMPK

**Figure 5:**
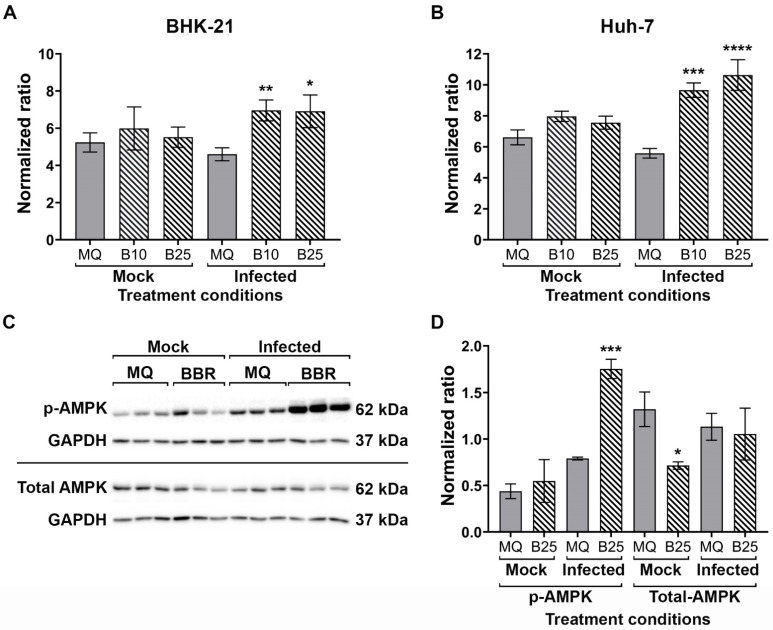
Effect of BBR on lipid droplets and AMPK. (**A**) BHK-21 and (**B**) Huh-7 cells were mock or DENV 2 infected followed by treatment with BBR or vehicle control (MQ). After 24 h cells were fixed and stained with ORO which was then eluted, and the absorbance was measured. Intensity levels were normalized to total protein. (**C**) BHK-21 cells were mock or DENV 2 infected followed by treatment with BBR or vehicle control (MQ). After 24 h cells were collected and proteins prepared and expression of p-AMPK, total AMPK and GAPDH were determined by western blot analysis. p-AMPK and total AMPK were detected on separate filters. (**D**) Band intensities were quantitated and normalized to GAPDH. Experiment was undertaken independently in triplicate. Error bars show ± S.E.M. * *p* ≤ 0.05, ** *p* ≤ 0.01 *** *p* ≤ 0.001, **** *p* ≤ 0.0001.

Several studies have shown the critical role of host cell lipids in the *Flavivirus* replication cycle [31], and particularly in virion assembly [32]. BBR has previously been investigated as a hypolipidemic agent, which can modify lipid homeostasis and metabolism [13,21], as well as interfere with lipogenesis and modulate oil droplet motility [22,33,34]. To determine whether BBR was exerting its antiviral activity through lipids, both BHK-21 and Huh-7 cells were either infected with DENV 2 or mock infected, and immediately after the infection period incubated with media containing BBR for 24 h. After this time cells were fixed, and stained with ORO, after which cells were washed, the ORO dye eluted with isopropanol and the intensity measured in a spectrophotometer. Parallel cells were used for protein extraction, and intensity data was normalized to protein concentration. Results (Figure 5A,B) showed that neither BBR treatment alone, nor infection alone altered the amount of cellular lipid in either cell line. Markedly however, the combination of infection and BBR treatment significantly increased cellular lipids in both cell lines (Figure 5A,B).

Adenosine-monophosphate activated kinase (AMPK) is an energy gauge protein which regulates fatty acid and cholesterol biogenesis that has been found to be impaired upon infection with flaviviruses [35], and so the status of this protein was investigated. BHK-21 cells were infected or mock infected and post-treated with BBR as previously described, before detection of phosphorylation status of AMPK (*p*-AMPK) and the level of total AMPK were determined by western blotting. The results showed a significant increase in p-AMPK in infected, BBR treated cells, that was not seen in either BBR treated only cells, or in infected cells not treated with BBR (Figure 5C,D). Levels of total AMPK were relatively consistent, albeit that treatment with BBR alone significantly reduced levels of AMPK (Figure 5C,D).

## 3. Discussion

DENV transmission occurs in more than 100 tropical and subtropical countries around the world [1] and this causes significant economic burdens both on nations and on the families of those affected [36,37]. While a commercial vaccine to protect against DENV infection was introduced recently, the association between vaccination and severe disease in flavivirus naïve vaccinees served to reduce the utility of this vaccine in controlling DENV [4,5]. Coupled with the lack of an effective vaccine, is the lack of any specific drug to treat DENV infection, and thus there is little to ameliorate the ongoing burden of DENV infections around the world.

In non-severe cases, the primary manifestation of DENV infection is a febrile illness [6], and many cultures have traditional medicines that are aimed to treat febrile diseases. Thus, in areas of high DENV transmission, it is possible that some of the traditional treatments have constituents with antiviral activities, and to date a number of compounds present in traditional remedies have been characterized for their anti-DENV activity [38]. BBR is an isoquinoline alkaloid that can be found in plants such as *Tinospora crispa* ([39] as cited in [10]), and studies have suggested that BBR may exert an antiviral activity against a number of viruses [15,16,17,18,19,20]. In Thailand, *Tinospora crispa* is known as “boraphet” and it has applications as an anti-fever and cooling agent [40], which prompted an investigation for possible anti-DENV activity.

The results have shown that BBR can affect DENV through two mechanisms. For the first mechanism, BBR was shown to have a direct inhibitory activity against the DENV virion, (as well as towards ZIKV and CHIKV), while in the second mechanism BBR exerted a cellular effect that served to reduce DENV production. A number of other natural compounds have been shown to exert a direct inhibitory effect on DENV, including baicalein [41,42], some steroidal saponins [43], betacyanins [44] and geraniin [45]. The mechanisms of action of such inhibitory compounds generally remains poorly characterized, but compounds could inhibit by binding directly to E protein and blocking receptor attachment, blocking the low pH mediated rearrangement of E protein required for endosomal membrane fusion, or by directly affecting the virion phospholipid membrane as has been recently shown for the lantibiotic Labyrinthopeptin A1 [46]. Studies with hepatitis C virus have suggested that BBR inhibited entry/attachment, possibly through an interaction with the HCV E2 protein [19].

In terms of cellular effects, BBR surprisingly increased the apparent level of infection as determined by flow cytometry under conditions of post-treatment. This was supported by western blot analysis that showed a significant increase in cellular E protein, but no concomitant increase in either NS1 or NS5 was observed. There was a slight, but non-significant increase in the level of DENV genome and NS1, both of which are normally exported from the cell. Collectively, these results would point to a failure of the virion production, either through a failure for the virion to form, or through a defect in viral egress. It also suggests that the level of the DENV genome is not a limiting factor determining virus production during DENV infection.

Previously, Varghese and colleagues have suggested that BBR exerts its antiviral effect in CHIKV infection through acting to reduce virus-induced MAPK signaling [17]. Consistent with their study, we found that BBR treatment resulted in a decrease of pERK1/2, but while their study showed that BBR treatment reduced the cellular level of the CHIKV structural E2 protein [17], our study clearly showed that BBR treatment increase cellular levels of the structural DENV E protein, suggesting that BBR exerts its effect through different pathways in DENV and CHIKV infection. Similarly, while several studies have suggested that BBR exerts its effect through acting on the NS5 protein [16,17,47] and reducing genome copy number replication [20], this was not observed in our study, again suggesting distinct mechanisms of action.

ERK1/2 activation has been previously shown to support viral replication [48], assembly [49] and trafficking [50] thus inhibition of ERK1/2 signaling could reduce viral levels in the supernatant, and led to accumulation of the DENV E protein in the cell, as seen here. In the secretory pathway, the exocyst complex composed of Sec3, Sec5, Sec6, Sec8, Sec10, Sec15, Exo70 and Exo84 is important for exocytosis of vesicles [28,29,51]. The Exo70 subunit of the exocyst complex has been reported to be a direct substrate of ERK1/2 phosphorylation, triggering complex formation for vesicle trafficking, and this pathway has been shown to be utilized by the DENV virion for egress [28,29,51]. However, while the ERK1/2 inhibitor U0126 effectively reduced both ERK1/2 activation and levels of Exo70, BBR only reduced ERK1/2 activation and had no effect on levels of Exo70, suggesting that BBR does not exert its effect through inhibition of DENV egress.

Several studies have shown the essential involvement of lipids and lipid metabolism during DENV infection [52,53]. BBR has been reported to modify lipid metabolism [23,34], possibly by exerting its activity through AMPK [22]. During DENV infection it is known autophagy is increased [54,55], and it is proposed that this results in depletion of cellular triglycerides stored in lipid droplets leading to increased energy available to support viral replication through increased β-oxidation [24]. In addition, it is proposed that altered lipid metabolism alters membrane fluidity to allow for the E protein embedded in membranes to efficiently envelope the nucleocapsid [56]. Our results showed that while neither infection alone, nor BBR treatment alone affected lipid droplets, the combination of infection and BBR treatment increased lipid droplets, suggesting increased lipid sequestration, and that this was associated with an increase in AMPK phosphorylation in infected/BBR treated cells. Thus, a possible mechanism is that the increased lipid sequestration in lipid droplets results in insufficient membrane permeability to allow virion formation, resulting in decreased virus production, and increased cellular E protein, as observed in this study. However, berberine has been shown to act through a number of other mechanisms including acting as an antioxidant and anti-inflammatory [57], as a repressor of DNA repair and replication [58], modulating noncoding RNAs, including miRNAs [59] and modulating autophagy [60], all of which could possibly impact how berberine affects viral replication, or its effects in other disease processes.

In conclusion, the isoquinoline alkaloid BBR which is a constituent in Thai and other regional traditional medicines has good potential for further development as an anti-DENV antiviral. In particular, this bioactive compound has already been widely studied for clinical applications against metabolic syndromes [13,21], suggesting its clinical utility. Markedly, BBR showed two distinct mechanisms of action, a direct virucidal activity, as well as a cellular effect that inhibited infectious virus production, possibly though an effect on lipid metabolism. Even though not further followed up in this study, BBR also showed good virucidal effects against two further arboviruses, namely ZIKV and CHIKV, suggesting the possibility of development as a broad spectrum anti-arboviral agent. Given that studies have shown an association between viral load in the blood and the severity of DENV infection [61], even a modest effect of BBR could have a significant impact upon disease severity.

## 4. Materials and Methods

### 4.1. Cell Culture

The human lung carcinoma cell line A549 (ATCC No. CCL-185), the baby hamster kidney cell line BHK-21 (ATCC No. CCL-10), the hepatocellular carcinoma cell line Huh-7 [62], the African green monkey kidney cell line Vero (ATCC No. CCL-81) and the Rhesus monkey kidney epithelial cell line LLC-MK2 (ATCC No. CCL-7) were cultured in in Dulbecco’s modified Eagle’s medium (DMEM; Gibco BRL, Gaithersburg, MD, USA) supplemented with 10% (*v*/*v*) or 5% (*v*/*v*) heat inactivated fetal bovine serum (FBS; Gibco BRL, Gaithersburg, MD, USA), 100 U/mL penicillin and 100 µg/mL streptomycin (Merk Millipore, Burlington, MA, USA) at 37 °C with 5% CO_2_. The insect *Aedes albopictus* cell line C6/36 (ATCC No. CRL-1660) was maintained in minimum essential media (MEM; GIBCO, Invitrogen, Grand Island, NY, USA) with 10% (*v*/*v*) FBS and 100 units/mL of penicillin and 100 μg/mL of streptomycin at 28 °C without CO_2_ supplementation.

### 4.2. Virus Propagation

DENV 2 (strain 16681) and ZIKV (strain MU1–2017 [63]) were propagated in C6/36 cells as described previously [63,64]. Briefly, C6/36 cells were infected with DENV 2 or ZIKV at a multiplicity of infection (MOI) of 0.1 and at the appearance of cytopathic effects (CPE) the supernatants were clarified by centrifugation, supplemented with FBS to a final concentration of 20% and stored at −80 °C. CHIKV (strain E1: 226V [65]) was propagated as previously described in Vero cells [66]. The viral titer for all viruses was determined by plaque assay either in Vero cells (ZIKV, CHIKV) or LLC-MK2 cells (DENV 2) as previously described [63,64,65]. Portions of the viral genome were amplified by reverse transcriptase-PCR (RT-PCR) followed by gel purification and sequencing of the PCR product to confirm the identity of each virus.

### 4.3. Compound Preparation

Berberine chloride was purchased from Sigma-Aldrich (633658; Sigma-Aldrich, St. Louis, MO, USA) and dissolved in either 100% dimethyl sulfoxide (DMSO) for virucidal experiments (Sigma, St. Louis, MO, USA) or milli-Q water (for all other experiments) to original stocks of 10 mM and 4 mM, respectively. The compound was further diluted in DMEM for specific experiments.

### 4.4. Cell Viability Assay

The MTT assay was used to determine cell cytotoxicity of BBR towards A549, HEK293T/17, Huh-7, HepG2 and BHK-21 cells. Briefly, cells were seeded in 96-well flat-bottomed plates at a density that allowed 80–90% confluence to be reached within 24 h. The culture media was removed before being replaced with normal growth media containing BBR at concentrations of 0.1–500 µM, or milli-Q water of the same volume. Cells were incubated for 24 h under standard conditions before addition of the thiazolyl blue tetrazolium bromide dye (Applichem GmBH, Darmstadt, Germany) Cells were incubated for a further 1.5 h before the formazan crystals were dissolved with DMSO, and the cell viability was calculated based on the absorbance values measured at 570 nm with 4 independent replicates (Beckman Coulter DX880 ST-52, Brea, CA, USA). Additionally, BHK-21 cells treated and maintained under the same conditions were observed under a light microscope at 10× magnification to observe their morphology, and images were captured.

### 4.5. Virucidal Activity Assay

Virucidal assays were undertaken as previously described [67]. Briefly, virus stocks of DENV 2, ZIKV and CHIKV were incubated directly at 37 °C in a water bath with the selected concentration of BBR (1–100 µM) in DMEM with no FBS, or with DMSO (0.01–1%) in DMEM with no FBS as a control. After 1 h, infectious viral titer was determined by plaque assay. Experiment was undertaken independently in triplicate with duplicate plaque assay [63,64,65].

### 4.6. Effect of BBR on DENV 2 Infection

BHK-21 cells were seeded and grown under standard conditions to achieve 70–80% confluence within 24 h. Three treatment protocols were investigated, namely (1) pre- and post-treatment in which the cells were treated for 2 h with BBR prior to DENV 2 (or mock) infection followed by 2 h infection in the absence of the drug, and washing before replacing the media with standard media containing BBR; (2) post-treatment in which the cells were infected with DENV 2 (or mock infected) for 2 h and subsequently cultured in standard culture media containing BBR after washing; and (3) pre-treatment in which cells were incubated for 2 h with BBR prior to infection, followed by washing and incubation in complete culture medium without BBR. After 24 h incubation under standard conditions, cells and supernatant were used as dictated by individual experiments. All experiments were undertaken independently in triplicate, with duplicate plaque assay where appropriate.

### 4.7. Flow Cytometry

Flow cytometry was undertaken essentially as previously described [68]. Briefly, infected and mock infected cells (treated with BBR as appropriate) were collected at 24 h post-infection (h.p.i) by centrifugation and were subsequently blocked with 10% normal goat serum (Gibco BRL, Gaithersburg, MD, USA) on ice before fixing with 4% paraformaldehyde (Merck KGaA, Darmstadt, Germany) in PBS-immunoflouresence assay (IFA) (150 mM NaCl, 50 mM NaH_2_PO_4_, 50 mM Na_2_HPO_4_, pH to 7.4) buffer for 20 min in the dark. Cells were then permeabilized with 0.2% Triton X-100 (OmniPur, Merck KGaA, Darmstadt, Germany) in PBS-IFA for 10 min in the dark at room temperature. Cells were then incubated with a 1:150 dilution of a pan-specific mouse anti-flavivirus E protein monoclonal antibody from hybridoma HB114 [69] in 0.75% BSA in PBS-IFA at 4 °C overnight. Between each step cells were washed with 0.75% bovine serum albumin (BSA; Capricorn Scientific GMbH, Ebsdorfergrund, Germany) in PBS-IFA. The following day, cells were incubated with a 1: 40 dilution of a goat anti-mouse IgG conjugated with fluorescein isothiocyanate (FITC; KPL, Gaitherburg, MD, USA) in 0.75% BSA in PBS-IFA for 1 h in the dark at room temperature. Finally, cells were resuspended in PBS-IFA before analysis on a BD FACSCalibur cytometer (Becton Dickinson, BD Biosciences, San Jose, CA, USA), using CELLQuest pro (Version 6.0) software. All samples were prepared and analyzed independently in triplicate.

### 4.8. Quantitative Real Time RT-PCR (qRT-PCR)

Cell lysates of infected or mock infected cells treated or not treated with BBR as appropriate were subjected to total RNA extraction using TRI reagent solution (Molecular Research Center, Inc., Cincinnati, OH, USA) according to the manufacturer’s protocol. After resuspending the RNA pellets with DEPC-treated water, RNA concentrations were measured using a Nanodrop ND-1000 UV-Vis spectrophotometer, and the final concentration of RNA was adjusted to 200 ng/μL using DEPC-treated water for further cDNA synthesis.

cDNA was generated from a known amount of RNA using Thermo Scientific RevertAid Reverse Transcriptase (Thermo Fisher Scientific, Waltham, MA, USA) and random hexamer primers (Thermo Fisher Scientific). Quantitative real time PCR was undertaken using cDNA and the KAPA SYBR FAST qPCR Kit 2X Master Mix (Kapa Biosystems Inc, Woburn, MA, USA) and NS1 specific primers (DENV-NS1-Fw: 5′-TGCTGACATGGGTTATTGGATAG-3′, DENV-NS1-Rv: 5′-ACTCCATTGCTCCACAGTGTGTG-3′) in a Mastercycler ep realplex real-time PCR machine. PCR cycle conditions used were 95 °C for 3 min, followed by 40 cycles of 95 °C for 10 s, 60 °C for 30 s and 72 °C for 20 s. Viral genome copy numbers were calculated against a standard curve generated from serial dilutions from 10^0^ to 10^10^ and read as copies/mL. PCR products of the standard were prepared using the FavorPrep GEL/PCR Purification Mini Kit [68] (Favogen BioTech, Ping-Tung, Taiwan) and subsequently diluted to various copy numbers following calculation using the Thermo Scientific Web.

### 4.9. Western Blotting

Mock infected or DENV 2 infected cells whether treated or untreated with BBR as appropriate were collected by trypsinization and proteins prepared as described previously [68]. Protein was separated by electrophoresis though 10% SDS polyacrylamide gels before transfer to 0.2 µm nitrocellulose membranes (GE Healthcare, Buckinghamshire, UK). Membranes were subsequently blocked with 5% skim milk in TBS/0.05% Tween 20 at room temperature and subsequently incubated with a pan-specific mouse anti-flavivirus E protein monoclonal antibody from hybridoma HB112 at a 1:500 dilution [69], a rabbit anti-dengue type 2 NS1 antibody at a 1:2000 dilution (PA5-27885, Thermo Scientific, Waltham, MA, USA), or a mouse anti-dengue type 2 NS5 monoclonal antibody at a 1:5000 dilution (MA5-17295, Thermo Scientific, Waltham, MA, USA) overnight at 4 °C. In addition, membranes were incubated for 1 h with a 1:5000 dilution of mouse anti-GAPDH monoclonal antibody (sc-32233; Santa Cruz Biotechnology Inc., Dallas, TX, USA). Secondary antibodies were either a horseradish peroxidase (HRP) conjugated goat anti-mouse IgG at a 1:5000 dilution (Sigma-Aldrich, St. Louis, MO, USA) or an HRP-conjugated goat anti-rabbit IgG at a 1:8000 dilution (Thermo Scientific, Waltham, MA, USA) as appropriate, and these were incubated with the membrane for 1 h at room temperature. The signals were developed with the Amersham ECL plus Western Blotting Detection Reagents (GE Healthcare, Chicago, IL, USA) and immediately captured using a visible western blot imaging system (Azure c400, Azure Biosystems, Inc., Dublin, CA, USA).

To detect phosphorylated proteins, cells were cultured and treated as above. Cells were directly lysed with Laemmli buffer containing 10% SDS. After extraction, proteins were immediately heated and stored at −30 °C until use. Western blot analysis was performed as above. Primary antibody used were a rabbit anti-Akt (pan)(C67E7) monoclonal antibody at a 1:1000 dilution (No. 4691, Cell Signaling Technology, Danvers, MA, USA), a rabbit- anti phospho-Akt (Ser473)(D9E) monoclonal antibody at a 1:2000 dilution (No. 4060, Cell Signaling Technology, Danvers, MA, USA), a rabbit anti-AMPKα polyclonal antibody at a 1:1000 dilution (No. 2532S, Cell Signaling Technology, Danvers, MA, USA), a rabbit anti phospho-AMPKα (T172) (40H9) monoclonal antibody at a 1:1000 dilution (No. 2535S, Cell Signaling Technology, Danvers, MA, USA), a rabbit anti p44/42 MAPK (ERK1/2)(137F5) monoclonal antibody at a 1:1000 dilution (No. 4695, Cell Signaling Technology, Danvers, MA, USA) a rabbit anti phospho-p44/42 MAPK (ERK1/2)(Thr202/Tyr204) polyclonal antibody at a 1:1000 dilution (No. 9101S, Cell Signaling Technology, Danvers, MA, USA), a rabbit anti-Exo70 polyclonal antibody at a 1:1000 dilution (MBS9611739, MyBioSource, Inc., San Diego, CA, USA) and a 1:5000 dilution of mouse anti-GAPDH monoclonal antibody (sc-32233; Santa Cruz Biotechnology Inc., Dallas, TX, USA). Secondary antibodies were a HRP-conjugated goat anti-rabbit IgG at a 1:8000–1:5000 dilution (Thermo Scientific, Waltham, MA, USA) and an HRP-conjugated goat anti-mouse IgG at a 1:5000 dilution (Sigma-Aldrich, St. Louis, MO, USA).

### 4.10. Oil Red O Staining (ORO)

ORO staining was undertaken essentially as previously described [70]. BHK-21 or Huh-7 cells were seeded at a density that allowed 80–90% confluence to be reached within 24 h, after which cells were mock infected or infected with DENV 2 for 2 h before being cultured for 24 h under standard conditions in complete media containing 10 or 25 µM of BBR or an equivalent volume of DMEM or milli-Q water. After incubation, cells were fixed with 10% formalin in PBS (Applichem, Munich, Germany) for 1 h. After fixing, cells were washed with 60% isopropanol prior to staining with filtered Oil Red O (Sigma-Aldrich, St. Louis, MO, USA) for 10 min, followed by washing with distilled water twice. ORO was eluted with 100% isopropanol and supernatant was transferred to a new tube and the absorbance was and measured at 490 nm (EZ Read 2000, Biochrom Ltd., Cambridge, UK).

### 4.11. Data Analysis

Band intensity analysis was undertaken using ImageJ 1.53a [71]. Statistical analysis was performed with GraphPad Prism 9 for Windows (GraphPad Software Inc., San Diego, CA, USA). Multiple comparisons were analyzed using analysis of variance (ANOVA), while single control comparisons were analyzed used an unpaired t-test. CC_50_, IC_50_ and EC_50_ were calculated using a non-linear fit dose response curve. Data is shown as mean *±* S.E.M. Significance is denoted by * *p* ≤ 0.05, ** *p* ≤ 0.01 *** *p* ≤ 0.001, **** *p* ≤ 0.0001.

## Data Availability

The data presented in this study are available in the article or the Supplementary Materials.

## Supplementary Materials

The following are available online, Supplemental Figure S1. Chemical structure of berberine, Supplemental Figure S2. Cytotoxicity of BBR towards several cell lines, Supplement Figure S3. BHK-21 morphology after BBR treatment, Supplemental Figure S4. Optimization of DENV infection, Supplemental Figure S5. EC_50_ calculation of BBR in DENV infection, Uncropped western blots.

## Sample Availability

Samples of the compounds are commercially available.
