# Supplementary material for: Berberine Inhibits Dengue Virus through Dual Mechanisms
Source: Molecules. 2021 Sep 10;26(18):5501. doi: 10.3390/molecules26185501 (PMC8470584; doi:10.3390/molecules26185501)
Supplement: Supplementary file 1 [file molecules-26-05501-s001.zip › molecules-1363266-supplementary.pdf]

molecules

MDPI

Supplemental materials

Berberine inhibits dengue virus through dual mechanisms

Thippayawan Ratanakomol[1], Sittiruk Roytrakul[2], Nitwara Wikan[1*], Duncan R. Smith[1*]

[1] Institute of Molecular Biosciences, Mahidol University, Salaya, 73170, Thailand; thippayawan.raa@student.mahidol.ac.th (T.R.); nitwara.wik@mahidol.ac.th (N.W.); duncan_r_smith@hotmail.com (D.R.S.)

[2] National Center for Genetic Engineering and Biotechnology (BIOTEC), National Science and Technology Development Agency, Pathum Thani, 12120, Thailand; (sittiruk@biotec.or.th)

* Correspondence: duncan_r_smith@hotmail.com or nitwara.wik@mahidol.ac.th; Tel.: +66-2800-3624-8

Supplemental Figure S1. Chemical structure of berberine.

Supplement Figure S2. Cytotoxicity of BBR towards several cell lines.

Supplement Figure S3. BHK-21 morphology after BBR treatment.

Supplemental Figure S4. Optimization of DENV infection.

Supplemental Figure S5. EC_{50} calculation of BBR in DENV infection.

Uncropped western blots

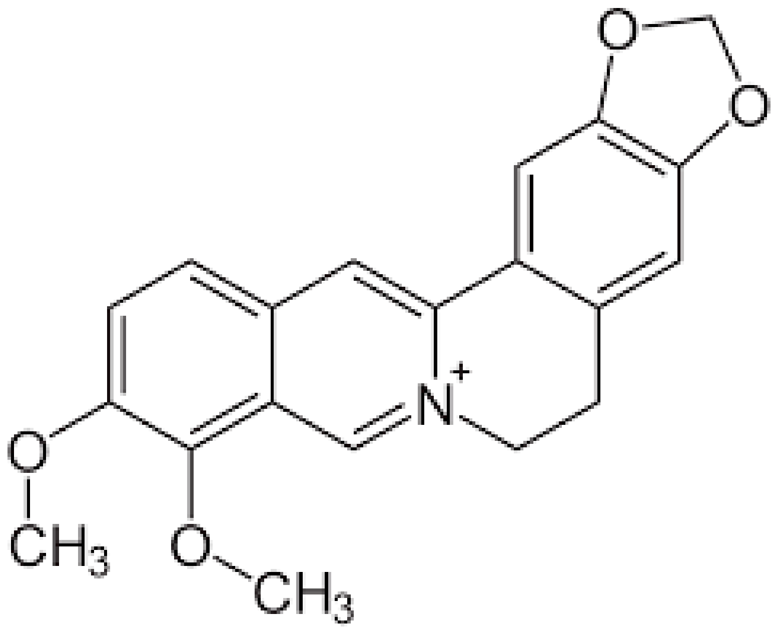

Supplemental Figure S1. Chemical structure of berberine.

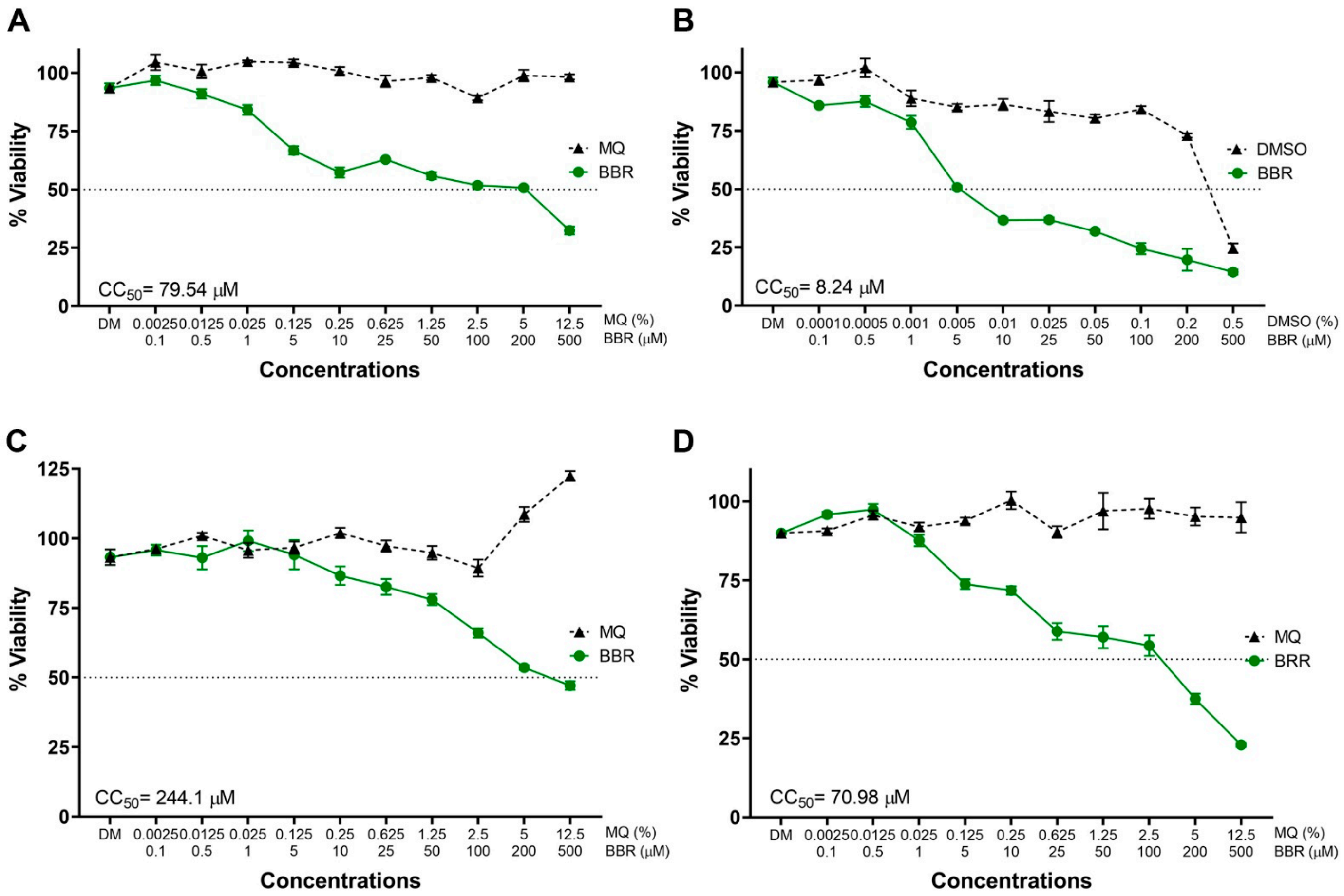

Supplemental Figure S2. Cytotoxicity of BBR towards several cell lines.

(A) A549, (B) HEK293T/17, (C) HepG2 and (D) Huh-7 cells were cultured in 96 well plates before being treated with different concentration of BBR or equivalent volume of MQ water in DMEM for 24 h before cell viability was determined by the MTT assay. Experiment was undertaken as 4 independent replicates. Errors bars show ± S.E.M. *p≤0.0001.

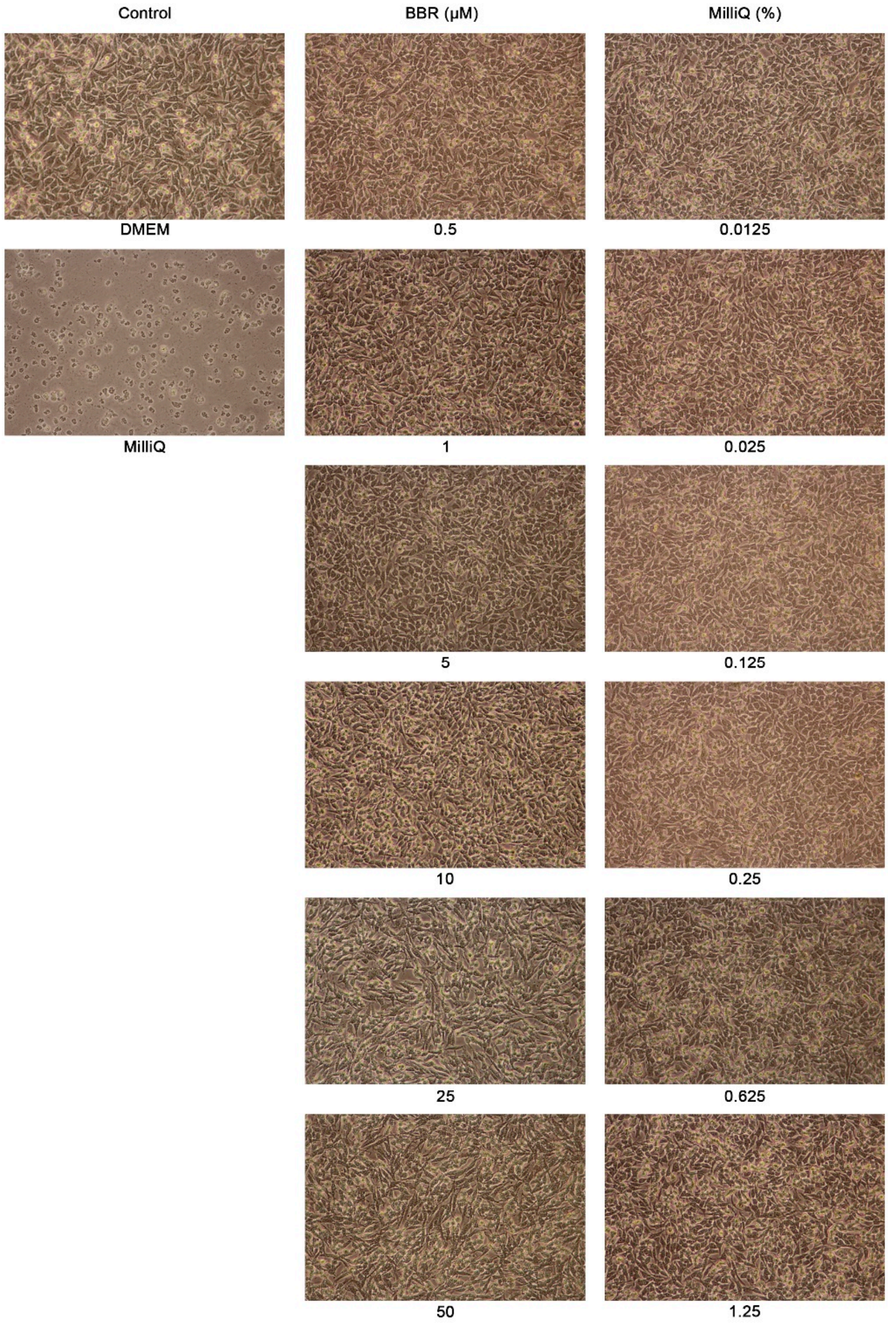

Supplemental Figure S3. BHK-21 morphology after BBR treatment.

BHK-21 cells were treated with BBR at concentrations ranging from 0.1 μM – 50 μM or an equivalent volume vehicle for 24 h before observation of cell morphology under an inverting microscope.

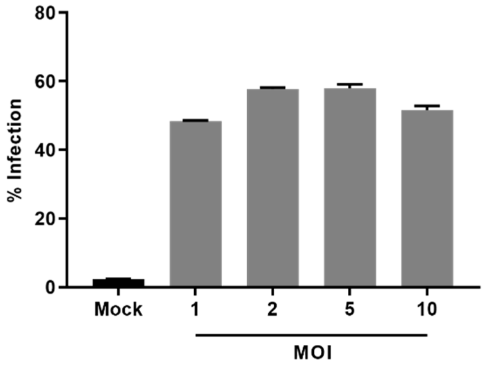

Supplemental Figure S4. Optimization of DENV infection. BHK-21 cells were infected with DENV 2 at different MOI, and afer 24 h, level of infection was determined by flow cytometry.

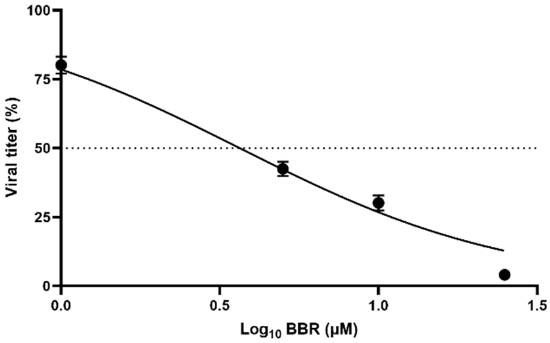

Supplemental Figure S5. EC_{50} calculation of BBR in DENV infection.
Virus titer data from Figure 2E was used to calculate the EC50 of BBR towards DENV under conditions of post treatment.

Uncropped western blots

Western blot of viral protein expression at 24 hours post- treatment

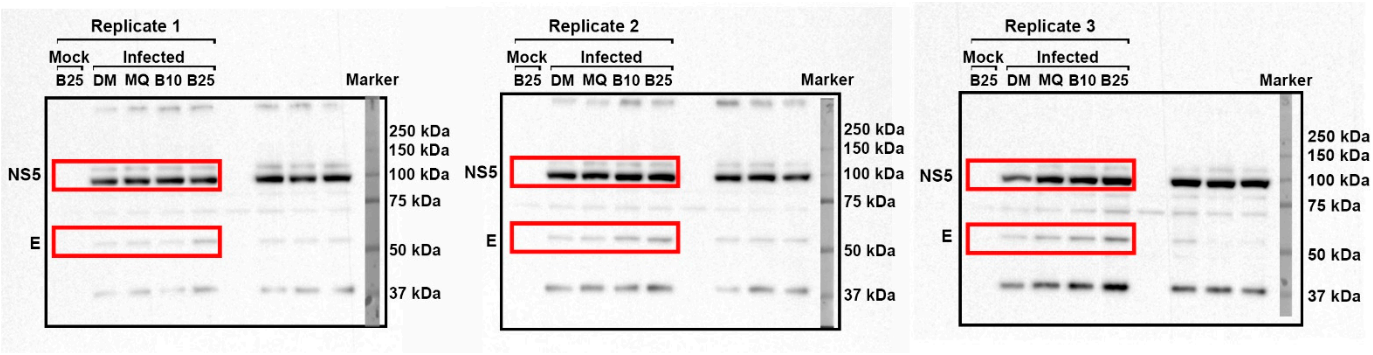

Full western blot membranes of NS5 and E proteins captured by Azure400 (Replicate 1-3 left-right), red boxes indicate bands presented in Fig 3D; NS5 and E proteins.

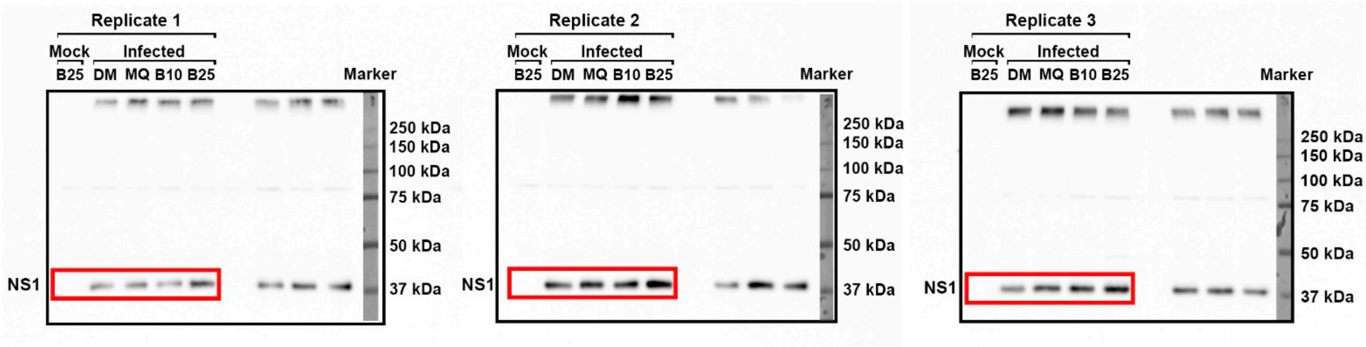

Full western blot membranes of NS1 protein captured by Azure400 (Rep 1-3 left-right), red boxes indicate bands presented in Fig 3D; NS1 protein.

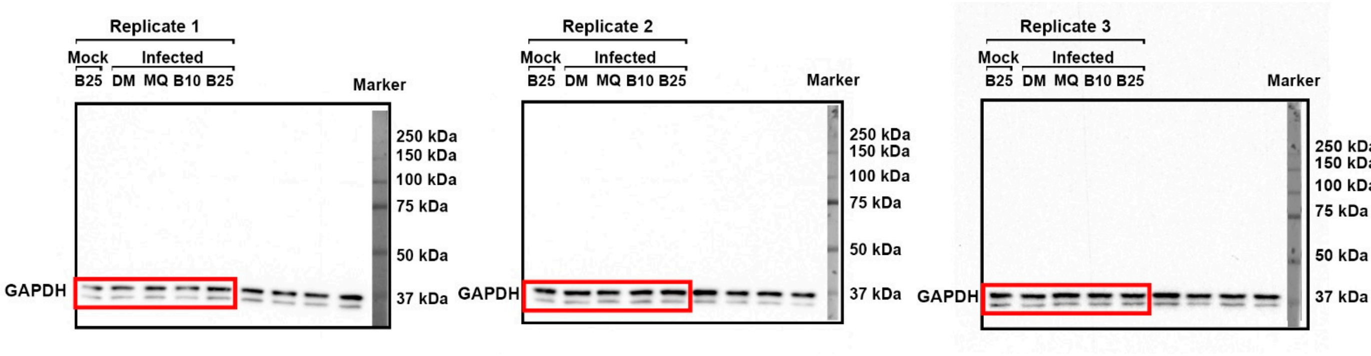

Full western blot membranes of GAPDH captured by Azure400 (Rep 1-3 left to right), red boxes indicate bands presented in Fig 3D; GAPDH.

Western blot of BBR effect on Erk1/2 and Exo70

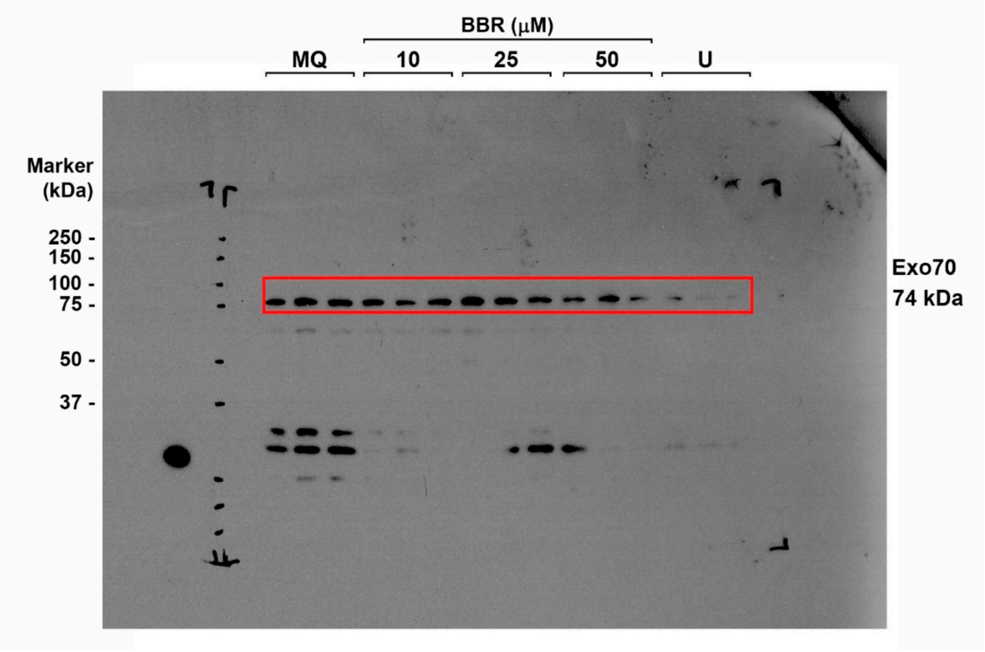

Full western blot membrane of Exo70 captured on X- ray film (Rep 1-3 were in consecutive lane), red box indicates bands presented on Fig 4A; Exo70.

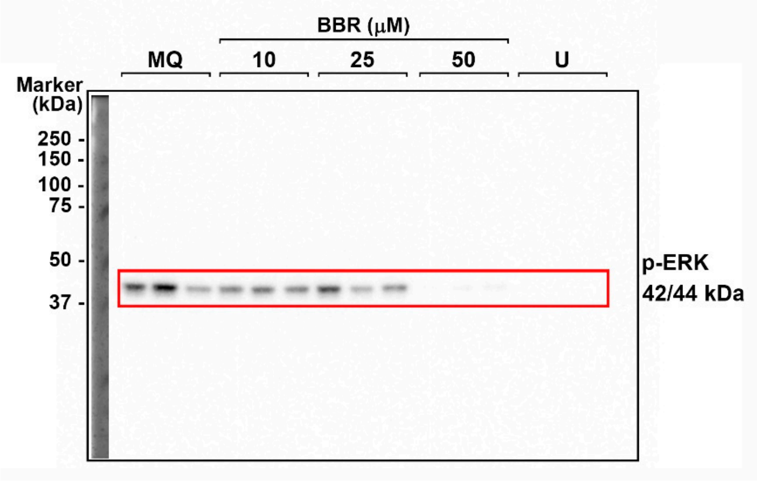

Full western blot membrane of p-ERK captured by Azure400 (Rep 1-3 were in consecutive lane), red boxes indicate bands presented in Fig 4A; p-ERK.

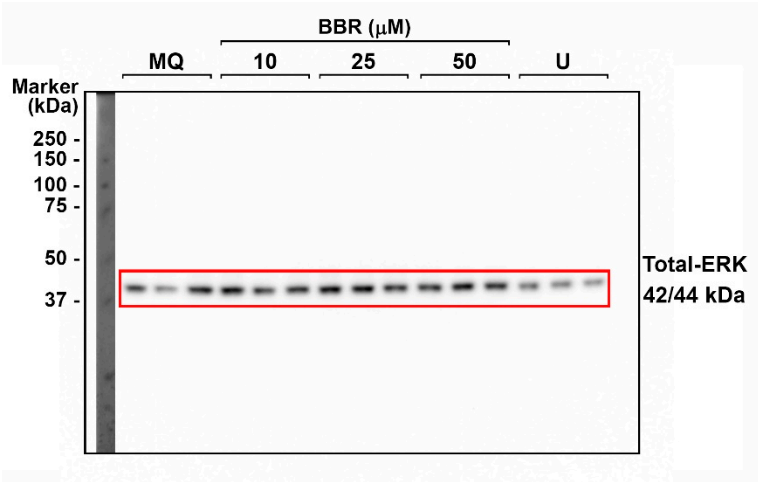

Full western blot membrane of total ERK captured by Azure400 (Rep 1-3 were in consecutive lane), red box indicates bands presented in Fig 4A; Total ERK.

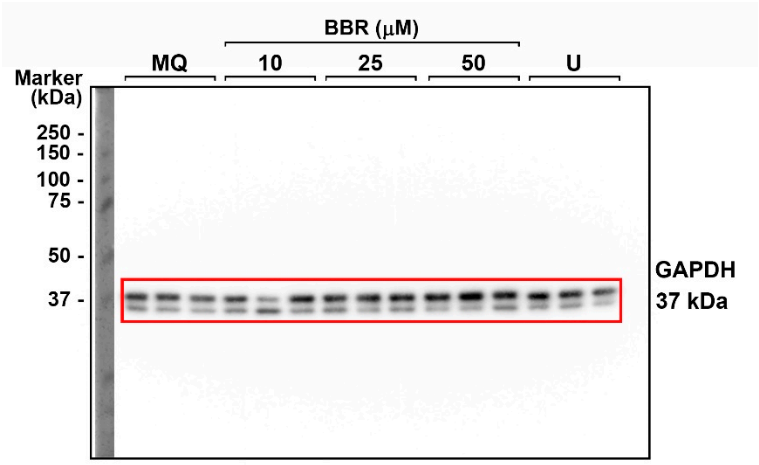

Full western blot membrane of GAPDH captured by Azure400 (Rep 1-3 were in consecutive lane), red box indicates bands presented in Fig 4A; GAPDH

Western blot of BBR effect on lipid droplets and AMPK

Note: Lane 15 left empty

p-AMPK and AMPK were performed on different membranes

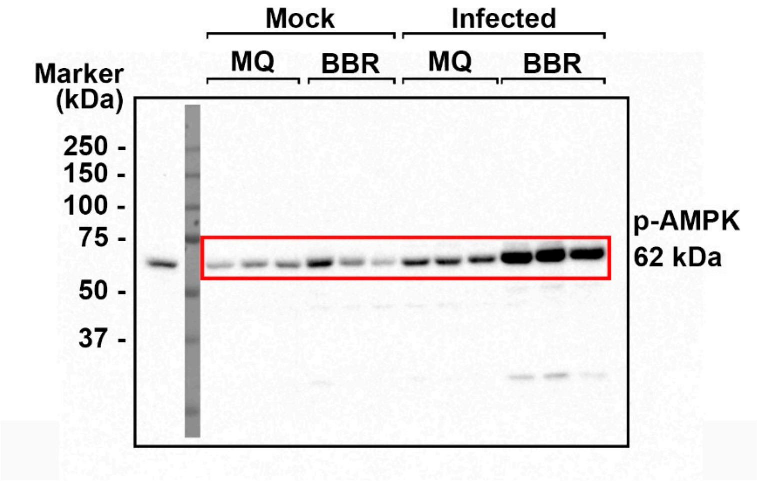

Full western blot membrane of p-AMPK captured by Azure400 (Rep 1-3 were in consecutive lane), red box indicates bands presented in Fig 5C; p- AMPK.

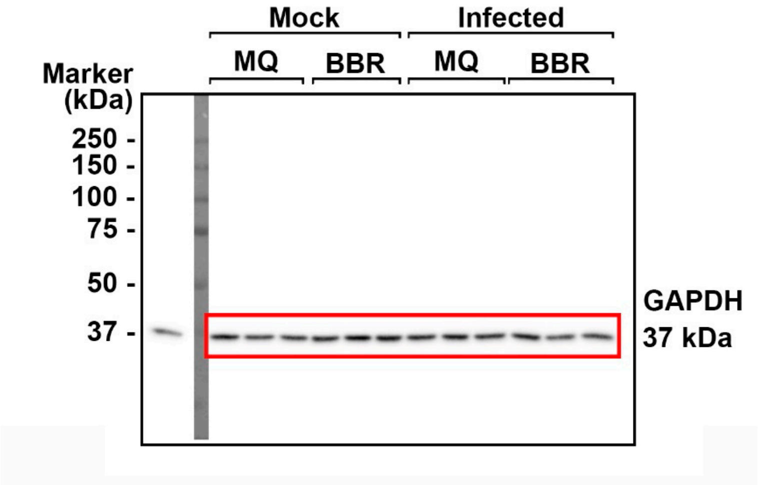

Full western blot membrane of GAPDH (from phospho- membrane) captured by Azure400 (Rep 1-3 were in consecutive lane), red box indicates bands presented in Fig 5C; GAPDH for p-AMPK.

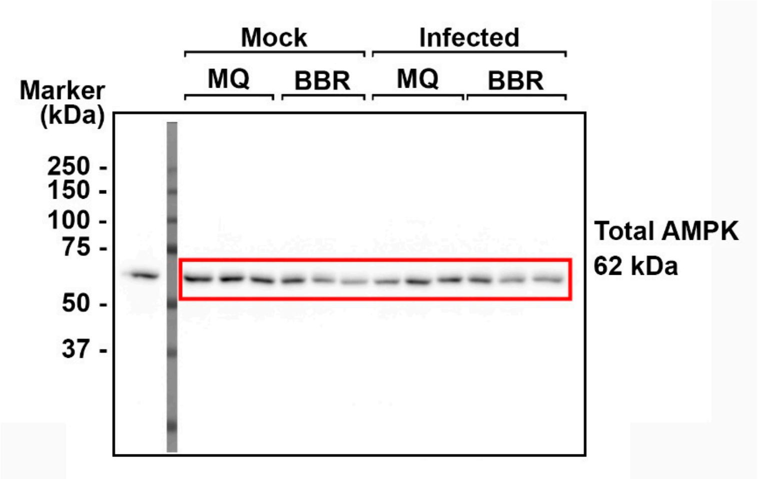

Full western blot membrane of total AMPK captured by Azure400 (Rep 1-3 were in consecutive lane), red box indicates bands presented in Fig 5C; total AMPK..

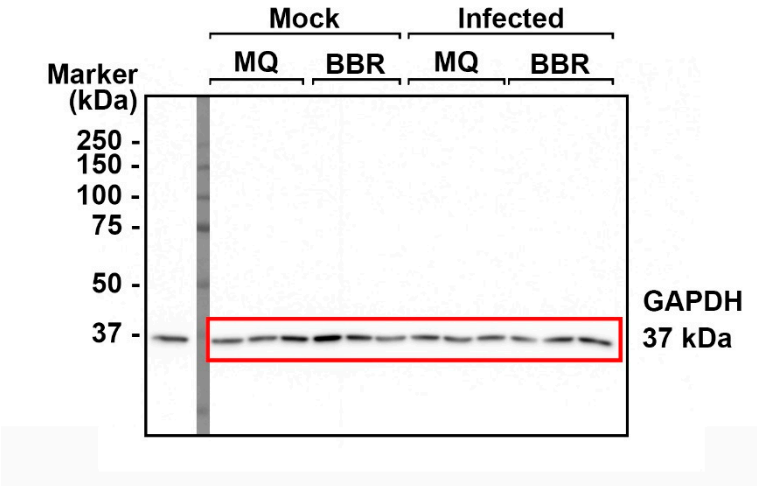

Full western blot membrane of GAPDH (total- membrane) captured by Azure400 (Rep 1-3 were in consecutive lane), red box indicates bands presented in Fig 5C; GAPDH for total AMPK.

.
